# Supplementary material for: Observation of exceptional points in reconfigurable non-Hermitian vector-field holographic lattices
Source: Nat Commun. 2016 Jul 18;7:12201. doi: 10.1038/ncomms12201 (PMC4960300; doi:10.1038/ncomms12201)
Supplement: Supplementary Information — Supplementary Figures 1-5, Supplementary Table 1, Supplementary Notes 1-4 and Supplementary References. [file ncomms12201-s1.pdf]

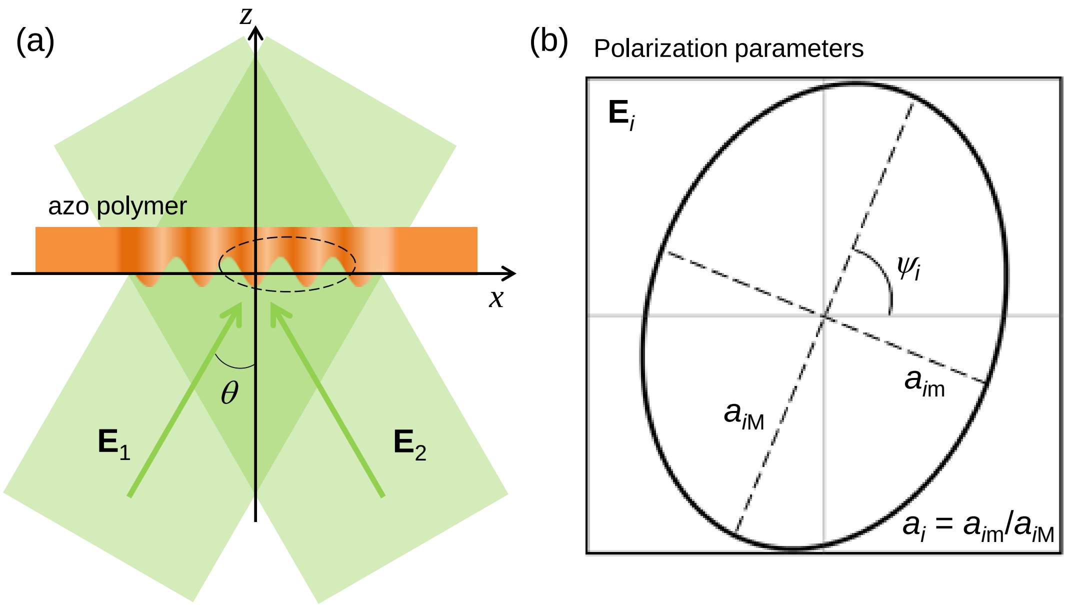

Supplementary Figure 1 | (a) Schematic illustration of two beam interference. (b) Illustration of polarization angle and ellipticity.

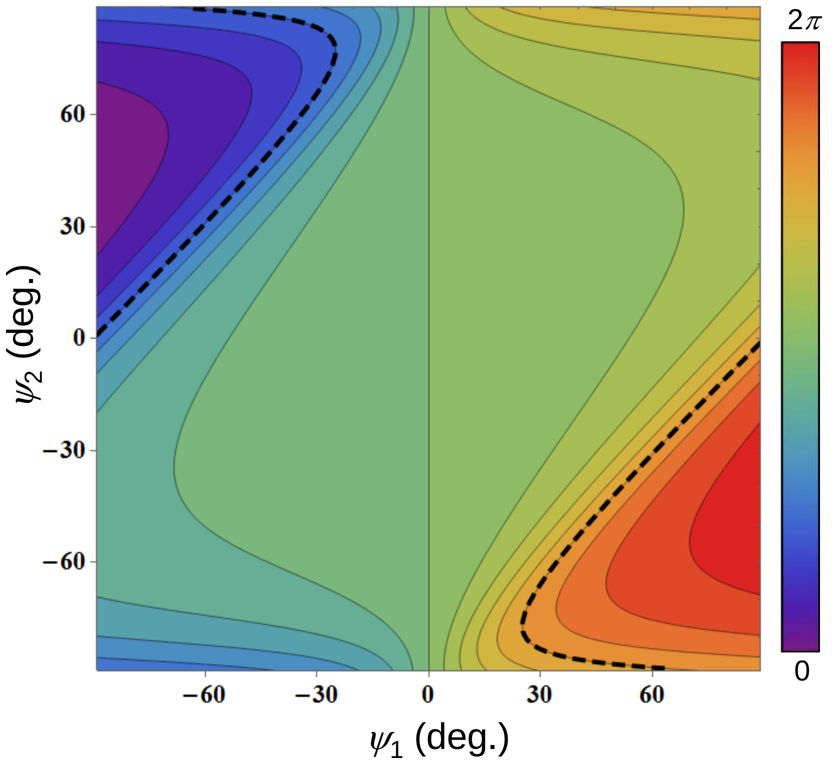

Supplementary Figure 2 | Phase difference parameter δ as a function of pump polarization angles ψ_1 and ψ_2 at $a_1 = 0$ and $a_2 = 0.23$. Black dashed lines indicate $\delta = \pi/2$ (near top-left corner) and $3\pi/2$ (near bottom-right corner).

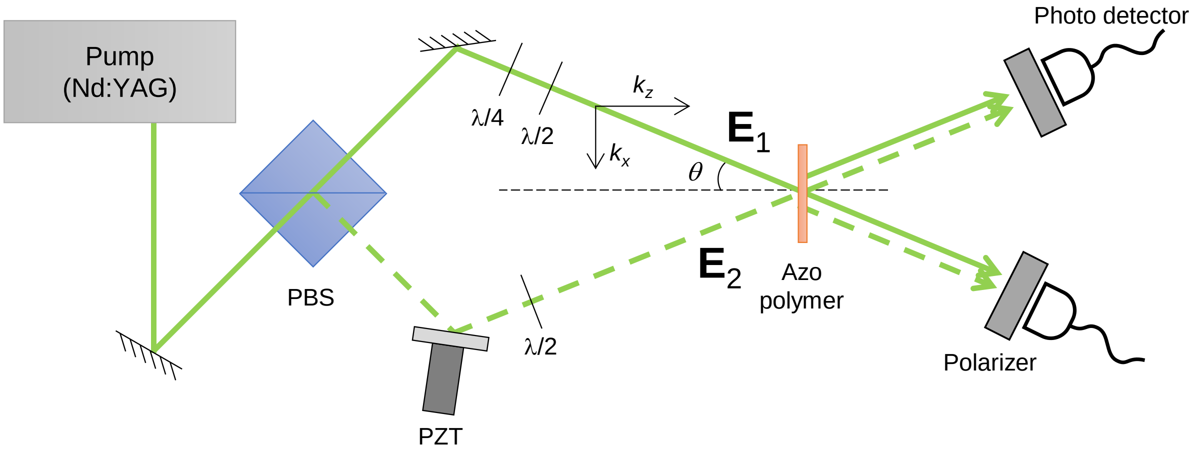

Supplementary Figure 3 | Schematic of two beam coupling system.

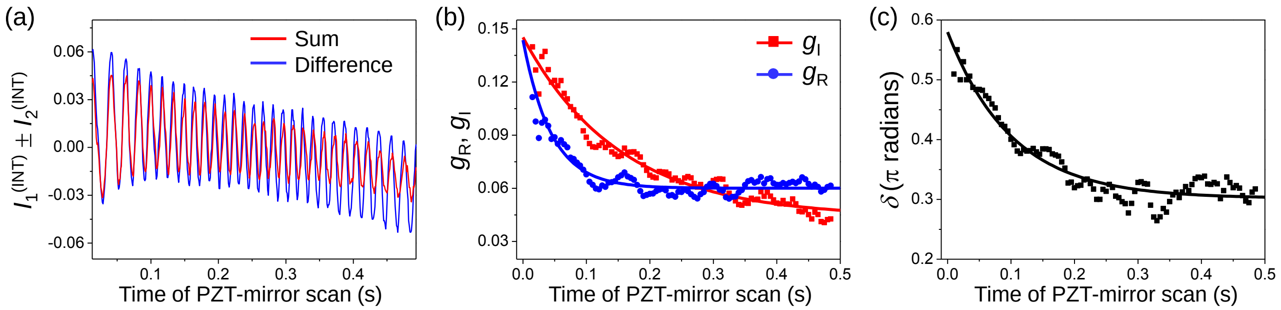

Supplementary Figure 4 | (a) Sum and difference of the two pump intensities during PZT scanning. (b) Change in modulation amplitude of the complex dielectric function $\Delta\varepsilon_R$ and $\Delta\varepsilon_I$, and (c) phase difference δ during PZT scanning.

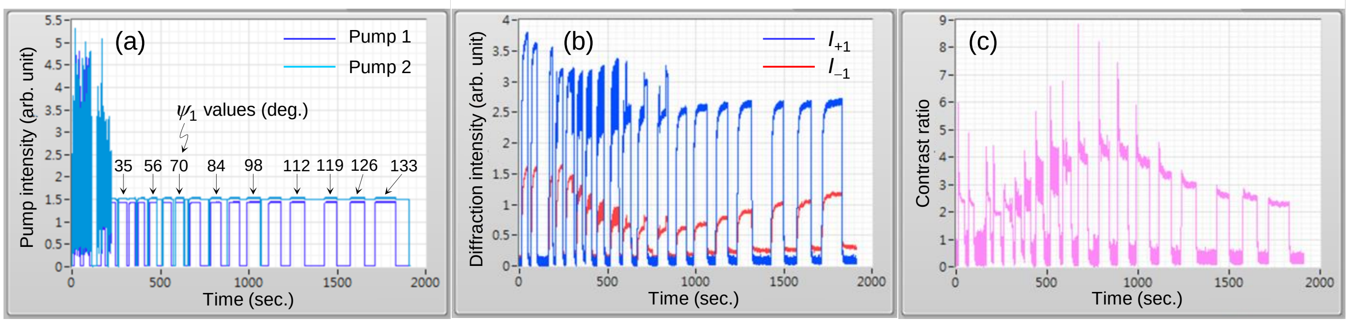

Supplementary Figure 5 | Reconfiguration of a complex lattice under multiple write-erase-rewrite cycles at a single sample spot. (a) Pump intensity profiles. (b) Diffraction intensity $I_{\pm 1}$ profiles. (c) Corresponding contrast ratio I_{+1}/I_{-1} profile. In this measurement, ψ_1 is tuned from 0° to 133° as indicated in (a) for each writing cycle. Other pump polarization parameters are fixed at e_1 = 1, e_2 = 0.67, and ψ_2 = 72.4°. The pump intensity is also fixed at 1.7 W cm^{-2}.

Mode description	Eigenvalue	Eigenvector
Anti-symmetric mode	$\alpha_1 = -Q\frac{k_0}{2\varepsilon_{\mathrm{avg}}^{1/2}}$	$\lvert \mathbf{u}_1 \rangle = N_1^{-1}\begin{bmatrix} \eta_{-1} \\ 0 \\ -\eta_{+1} \end{bmatrix}$
Symmetric mode	$\alpha_2 \simeq -\left(Q + \frac{2\eta_{+1}\eta_{-1}}{Q}\right)\frac{k_0}{2\varepsilon_{\mathrm{avg}}^{1/2}}$	$\lvert \mathbf{u}_2 \rangle \simeq N_2^{-1}\begin{bmatrix} \eta_{-1} \\ -2\eta_{+1}\eta_{-1}/Q \\ \eta_{+1} \end{bmatrix}$
Zero-order dominant mode	$\alpha_3 \simeq \frac{2\eta_{+1}\eta_{-1}}{Q}\frac{k_0}{2\varepsilon_{\mathrm{avg}}^{1/2}}$	$\lvert \mathbf{u}_3 \rangle \simeq N_3^{-1}\begin{bmatrix} \eta_{-1} \\ Q \\ \eta_{+1} \end{bmatrix}$

Supplementary Table 1. Eigenvalues and eigenvectors of **H**.

Supplementary Note 1: Expressions for real and imaginary sub-grating profiles

The azobenzene dyes have two isomer phases referred to as 'trans' and 'cis' molecules.[1,2] The trans phase is more stable than the cis phase and the trans molecules preferentially absorb photons with polarization parallel to the molecular long-axis.[3,4] Consequently, the isomerization processes under a polarized pump field results in net stationary population of aligned trans molecules with their long axis perpendicular to the pump polarization. Anisotropic absorption due to this process is called angular hole burning,[5-7] and is the dominant mechanism responsible for the formation of the imaginary sub-grating in our method. Considering angular hole burning in our excitation configuration (see Fig. 1a in the main text), we model the modulation of the imaginary dielectric function as:

$$\Delta\varepsilon_{\mathrm{I}} = C_{\mathrm{I}} P \cos(2\varphi)\left(1-a^2\right), \tag{1}$$

where P is the pump intensity, C_{I} is an empirical constant, φ is the angle of the major axis of the polarization ellipse of $\mathbf{E}_{\mathrm{P}}$ from the probe polarization that experiences the modulated absorption, and a is the ratio of the minor to major axis of the polarization ellipse of $\mathbf{E}_{\mathrm{P}}$. Note that a = 1 for circular polarization and a = 0 for linear polarization. For a TE-polarized probe having an electric field oscillating along the y axis, equation (1) is written as:

$$\Delta\varepsilon_{\mathrm{I}} = C_{\mathrm{I}} 2\left(1+\frac{\left|E_{\mathrm{P}x}{}^2+E_{\mathrm{P}y}{}^2\right|}{\left|E_{\mathrm{P}x}\right|^2+\left|E_{\mathrm{P}y}\right|^2}\right)^{-1}\left(\left|E_{\mathrm{P}x}\right|^2-\left|E_{\mathrm{P}y}\right|^2\right). \tag{2}$$

For a TM-polarized probe having an electric field oscillating along the x axis, the same relation is valid with the last term on the right-hand side replaced with $|E_{\mathrm{P}y}|^2-|E_{\mathrm{P}x}|^2$. Introducing new polarization state parameters $\alpha = \tan^{-1}(|E_{\mathrm{P}y}/E_{\mathrm{P}x}|)$ and $\varphi_{xy} = \arg(E_{\mathrm{P}x})-\arg(E_{\mathrm{P}y})$, equation (2) can be alternatively expressed by equation (2) in the main text. For $\mathbf{E}_1$ and $\mathbf{E}_2$ of which excitation and polarization configurations are shown in Supplementary Figs. 1(a) and 1(b), respectively, we find an expression for $\mathbf{E}_{\mathrm{P}}$ as:

$$\begin{aligned}\mathbf{E}_{\mathrm{P}} &= \mathbf{E}_1+\mathbf{E}_2\\ &= E_0\begin{pmatrix}\frac{1}{\sqrt{1+a_1^2}}(\cos\psi_1+a_1 i\sin\psi_1)\cos\theta e^{ik_x x}+\frac{1}{\sqrt{1+a_2^2}}(\cos\psi_2+a_2 i\sin\psi_2)\cos\theta e^{-ik_x x}\\ \frac{1}{\sqrt{1+a_1^2}}(\sin\psi_1-a_1 i\cos\psi_1)e^{ik_x x}+\frac{1}{\sqrt{1+a_2^2}}(\sin\psi_2-a_2 i\cos\psi_2)e^{-ik_x x}\\ -\frac{1}{\sqrt{1+a_1^2}}(\cos\psi_1+a_1 i\sin\psi_1)\sin\theta e^{ik_x x}+\frac{1}{\sqrt{1+a_2^2}}(\cos\psi_2+a_2 i\sin\psi_2)\sin\theta e^{-ik_x x}\end{pmatrix}e^{i(k_z z-\omega t)}.\end{aligned} \tag{3}$$

Substituting equation (3) into equation (2), we obtain the periodic profile of $\Delta\varepsilon_{\mathrm{I}}(x)$ for given pump fields.

Now, we describe the real dielectric function modulation due to migration of azobenzene-polymer complexes[7,8] due to an optical gradient force acting along the axis of the periodicity. The time-averaged optical gradient force is written as:

$$\mathbf{F}(\mathbf{r}) = \left\langle \mathbf{P}(\mathbf{r},t)\cdot\nabla\mathbf{E}_{\mathrm{P}}(\mathbf{r},t)\right\rangle_t = \frac{1}{2}\varepsilon_0\chi\left\langle \mathbf{E}_{\mathrm{P}}(\mathbf{r},t)\cdot\nabla\mathbf{E}_{\mathrm{P}}(\mathbf{r},t)\right\rangle_t, \tag{4}$$

where $\mathbf{P} = \varepsilon_0\chi\mathbf{E}_{\mathrm{P}}$ is the induced dipole moment density, $\langle\ldots\rangle_t$ implies time-averaging of the argument, ε_0 and χ denote the permittivity of vacuum and the susceptibility of the azo polymer, respectively. Molecular migration due to the periodic gradient force induces a surface-relief grating (SRG) that represents a modulation in the real dielectric function. In more detail, the azo molecules near positive or negative local maxima migrate in the $\pm x$ directions. The result of this process is modulation of the real dielectric function modeled as:

$$\Delta\varepsilon_{\mathrm{R}} = \frac{C_{\mathrm{R}}}{\varepsilon_0}\frac{\partial}{\partial x}F_x, \tag{5}$$

where C_{R} is an empirical constant. Substituting equation (4) into equation (5) yields equation (1) of the main text.

Applying the expression for the pump field in equation (3) to equations (2) and (5), we calculate the phase parameter δ in equation (3) of the main text as a function of pump polarization angles ψ_1 and ψ_2. Supplementary Fig. 2 shows a ψ_1-ψ_2 map of δ for one exemplary combination of a_1 = 0 and a_2 = 0.23. We clearly confirm that an arbitrary δ value in the full range of [0, 2π] is accessible by adjusting ψ_1 and ψ_2. The contours indicated by black dashed curves represent $\delta = \pi/2$ or $3\pi/2$ for the exact PT-symmetry condition. Finding optimal pump polarization conditions for experiments employing different polarization control schemes, one can generate a ψ_1-ψ_2 map of δ for other combinations of a_1 and a_2.

Supplementary Note 2: Hamiltonian formulation, vector-field skewness, and contrast ratio in complex photonic lattices

Hamiltonian formulation

Since $\Delta\varepsilon_R$ and $\Delta\varepsilon_I$ in our experimental configuration are of the order of 10^{-2} or 10^{-1}, we take the slowly varying envelope approximation to mathematically describe the evolution of the probe electric field **E** in the generated complex lattices. Assuming $\mathbf{E}(x,z) = \mathbf{e}_y\tilde{E}(x,z)\exp(ikz)$ at the angular frequency ω with $\mathbf{e}_y$ being the unit vector along the y axis, $\varepsilon(x) = \varepsilon_{\text{avg}}+\Delta\varepsilon(x)$, and $k = \varepsilon_{\text{avg}}^{1/2}\omega/c$, the frequency-domain Maxwell's equations yield the following wave equation:

$$-2ik\frac{\partial\tilde{E}(x,z)}{\partial z} = \frac{\partial^2\tilde{E}(x,z)}{\partial x^2} + \Delta\varepsilon(x)k_0{}^2\tilde{E}(x,z)\,. \tag{6}$$

Here, we assume the coordinate system illustrated in Fig. 1a of the main text. Using Fourier decomposition and the Floquet-Bloch theorem, we express $\Delta\varepsilon(x)$ and $\tilde{E}(x,z)$ as:

$$\Delta\varepsilon(x) = \sum_{m=-\infty}^{\infty}\eta_m\exp(imKx)\,, \tag{7}$$

$$\tilde{E}(x,z) = \sum_{m=-\infty}^{\infty}A_m(z)\exp(imKx)\,, \tag{8}$$

where $K = 2\pi/\Lambda$ is the grating wavenumber. By substituting these two expressions into equation (6), we obtain a coupled-mode equation

$$-2ik\frac{\partial A_m(z)}{\partial z} = -(mK)^2A_m(z) + k_0{}^2\sum_{l=-\infty}^{\infty}\eta_{m-l}A_l(z)\,. \tag{9}$$

Introducing a column vector $|\mathbf{A}(z)\rangle \equiv [\ldots\ A_m(z)\ \ldots]^{\mathrm{T}}$, equation (9) is expressed as a Hamiltonian matrix equation:

$$-i\frac{d}{dz}\left|\mathbf{A}(z)\right\rangle = \mathbf{H}\left|\mathbf{A}(z)\right\rangle\,. \tag{10}$$

The Hamiltonian matrix elements are given by

$$H_{nm} = \frac{k_0{}^2}{2k}\left[\eta_{n-m} - (mK/k_0)^2\delta_{nm}\right]. \tag{11}$$

Skewness parameters

In a first-harmonic complex grating, the complex index modulation $\Delta\varepsilon(x)$ can be written as:

$$\begin{aligned}\Delta\varepsilon(x) &= \Delta\varepsilon_0\left[(1-\xi)\cos(Kx) + i\xi\cos(Kx-\delta)\right]\\ &= \eta_{+1}e^{iKx} + \eta_{-1}e^{-iKx},\end{aligned} \tag{12}$$

where η_{+1} and η_{-1} represent the p = +1 and –1 diffraction order potentials, respectively. In the three-channel approximation, which includes the p = –1, 0, and +1 diffraction orders as allowed propagating modes, the Hamiltonian **H** from equations (10) and (11) simplifies to:

$$\mathbf{H} = \frac{k_0}{2\varepsilon_{\text{avg}}^{1/2}}\begin{pmatrix}-Q & \eta_{-1} & 0\\ \eta_{+1} & 0 & \eta_{-1}\\ 0 & \eta_{+1} & -Q\end{pmatrix}, \tag{13}$$

where $Q = (K/k_0)^2$. Solving an eigenvalue problem $\mathbf{H}|\mathbf{u}_\nu\rangle = \alpha_\nu|\mathbf{u}_\nu\rangle$, we obtain eigenvalues and eigenvectors in the small modulation limit ($Q >> \eta_{\pm1}$) as listed in Table 1 below. Note in Table 1 that N_ν denotes a normalization factor.

Now, we define the skewness parameters as:

$$c_{\nu\mu} \equiv \left|\left\langle\mathbf{u}_\nu\middle|\mathbf{u}_\mu\right\rangle\right|. \tag{14}$$

The skewness parameters by their definition yield a positive real value between 0 for orthogonal eigenvectors and 1 for eigenvectors merging at an exceptional point. From the above definition, we find expressions of the skewness parameters as:

$$c_{12} = \frac{\left| |\eta_{+1}|^2 - |\eta_{-1}|^2 \right|}{\sqrt{\left| \left(|\eta_{+1}|^2 + |\eta_{-1}|^2 \right)\left(|\eta_{+1}|^2 + |\eta_{-1}|^2 + (2|\eta_{+1}\eta_{-1}|/Q)^2 \right) \right|}} \simeq \frac{\left| |\eta_{+1}|^2 - |\eta_{-1}|^2 \right|}{|\eta_{+1}|^2 + |\eta_{-1}|^2}, \tag{15}$$

$$c_{23} = \frac{\left| |\eta_{+1}|^2 + |\eta_{-1}|^2 - 2\eta_{+1}\eta_{-1} \right|}{\sqrt{\left| \left(|\eta_{+1}|^2 + |\eta_{-1}|^2 + (2|\eta_{+1}\eta_{-1}|/Q)^2 \right)\left(|\eta_{+1}|^2 + |\eta_{-1}|^2 + Q^2 \right) \right|}} \simeq \frac{\left| |\eta_{+1}|^2 + |\eta_{-1}|^2 - 2\eta_{+1}\eta_{-1} \right|}{Q\sqrt{|\eta_{+1}|^2 + |\eta_{-1}|^2}} \simeq 0\,, \tag{16}$$

$$c_{31} = \frac{\left| |\eta_{+1}|^2 - |\eta_{-1}|^2 \right|}{\sqrt{\left| \left(|\eta_{+1}|^2 + |\eta_{-1}|^2 \right)\left(|\eta_{+1}|^2 + |\eta_{-1}|^2 + Q^2 \right) \right|}} \simeq \frac{\left| |\eta_{+1}|^2 - |\eta_{-1}|^2 \right|}{Q\sqrt{|\eta_{+1}|^2 + |\eta_{-1}|^2}} \simeq 0\,. \tag{17}$$

We note that $c_{23} \approx c_{31} \approx 0$, implying that $|\mathbf{u}_3\rangle$ is acceptably orthogonal to both $|\mathbf{u}_1\rangle$ and $|\mathbf{u}_2\rangle$. In contrast, $|\mathbf{u}_1\rangle$ and $|\mathbf{u}_2\rangle$ have a significant projection on each other provided that $|\eta_{+1}| \neq |\eta_{-1}|$, which occurs generally in complex lattices.

Contrast ratio in terms of skewness parameters

For normal incidence, the initial state is given by $|\mathbf{A}(0)\rangle = [0\ E_0\ 0]^T$, and the diffracted wave at $z = d$ in the short-propagation limit $k_0 d << 1$ is determined by evaluating equation (10) yielding:

$$\begin{aligned} |\mathbf{A}(d)\rangle &= \exp[i\mathbf{H}d]\,|\mathbf{A}(0)\rangle \\ &\simeq \left(\mathbf{I} + i\mathbf{H}d - \mathbf{H}^2 d^2\right)|\mathbf{A}(0)\rangle \\ &\simeq E_0 \begin{pmatrix} i\rho\eta_{-1} \\ 1 - 2\rho^2\eta_{+1}\eta_{-1} \\ i\rho\eta_{+1} \end{pmatrix}, \end{aligned} \tag{18}$$

where $\rho \equiv k_0 d/2\varepsilon_{\text{avg}}^{1/2}$. In the output vector, the first and third components represent left and right diffracted waves, respectively. From equation (18), we find the diffracted intensities into the $p = \pm 1$ diffraction orders as $I_{\pm 1} = \rho^2|\eta_{\pm 1}|^2$. Associating equation (15) for the skewness parameter c_{12} with the diffracted intensities, a relationship to the contrast ratio $\Gamma = I_{+1}/I_{-1}$ is obtained:

$$c_{12} \simeq \left| \frac{\Gamma - 1}{\Gamma + 1} \right|. \tag{19}$$

Equation (6) in the main text is obvious from equation (19).

Supplementary Note 3: Phase-shifting interferometry for complex dielectric function measurement

Our measurement method is based on the approach used in Refs. 9 and 10. Briefly explaining the method with reference to the schematic illustration in Supplementary Fig. 3, two coherent beams from a frequency doubled Nd:YAG laser are incident on the azo polymer film at an angle of incidence θ. $\mathbf{E}_1$ is elliptically polarized and $\mathbf{E}_2$ is linearly polarized. They form an interference pattern in azo polymer which generates a complex refractive index grating. The in-plane wavevector component of the incident field satisfies the Bragg condition $k_x = K/2$ with the grating wavenumber being $K = 2\pi/\Lambda$. Therefore, the electric field in equation (8) becomes

$$\tilde{E}(x,z) = \sum_{m=-\infty}^{\infty} A_m(z)\exp\left[i\left(mK + k_x\right)x\right] = \sum_{m=-\infty}^{\infty} A_m(z)\exp\left[i\left(m + \frac{1}{2}\right)Kx\right]. \tag{20}$$

By substituting equation (20) into equation (6), we obtain a coupled-mode equation for the m-th harmonic component as

$$-2ik\frac{\partial A_m(z)}{\partial z} = -\left[\left(m + \frac{1}{2}\right)K\right]^2 A_m(z) + k_0{}^2 \sum_{l=-\infty}^{\infty} \eta_{l-m} A_l(z)\,. \tag{21}$$

The Hamiltonian matrix elements in this case are given by:

$$H_{nm} = \frac{k_0}{2\varepsilon_{\text{avg}}^{1/2}}\left[\eta_{n-m} - \left(\frac{(m+1/2)}{k_0}K\right)^2 \delta_{nm}\right]. \quad (22)$$

For two incident beams, we consider a two-channel problem including $m = -1$ and 0 with the Hamiltonian given by

$$\mathbf{H} = \frac{k_0}{2\varepsilon_{\text{avg}}^{1/2}}\begin{bmatrix} -\frac{1}{4}(K/k_0)^2 & \eta_{-1} \\ \eta_{+1} & -\frac{1}{4}(K/k_0)^2 \end{bmatrix}. \quad (23)$$

The output fields for two input beams are obtained by solving

$$\begin{aligned} \left|\mathbf{A}(d)\right\rangle &= \exp\left(i\mathbf{H}d\right)\left|\mathbf{A}(0)\right\rangle \simeq \left(1+i\mathbf{H}d\right)\left|\mathbf{A}(0)\right\rangle \\ &= \begin{pmatrix} 1-i\rho Q/4 & i\rho\eta_{-1} \\ i\rho\eta_{+1} & 1-i\rho Q/4 \end{pmatrix}\left|\mathbf{A}(0)\right\rangle \\ &\simeq \begin{pmatrix} 1 & i\rho\eta_{-1} \\ i\rho\eta_{+1} & 1 \end{pmatrix}\begin{pmatrix} E_1 e^{i\Phi} \\ E_2 \end{pmatrix} \end{aligned} \quad (24)$$

with the initial condition $|\mathbf{A}(0)\rangle = [E_1e^{i\Phi}\ E_2]^{\text{T}}$. Here, we assume $\rho << 1$ for an optically thin azo polymer film. Shifting the interference pattern by moving a mirror on a piezoelectric translation stage in one branch of the pump fields, changes the initial phase Φ. The Φ-dependent output field is then obtained as

$$\begin{aligned} A_1(d) &= E_1 e^{i\Phi} + i\rho\eta_{-1}E_2 \\ A_2(d) &= E_2 + i\rho\eta_{+1}E_1 e^{i\Phi} \end{aligned}, \quad (25)$$

with their intensities given by:

$$\begin{aligned} I_1 &= \left|A_1(d)\right|^2 = \left|E_1\right|^2 + \rho^2\left|\eta_{-1}E_2\right|^2 + 2E_1E_2\rho\,\text{Re}\left\{-i\eta_{-1}{}^* e^{i\Phi}\right\} \\ I_2 &= \left|A_2(d)\right|^2 = \underbrace{\left|E_2\right|^2 + \rho^2\left|\eta_{+1}E_1\right|^2}_{I^{(\text{DC})}} + \underbrace{2E_1E_2\rho\,\text{Re}\left\{i\eta_{+1}e^{i\Phi}\right\}}_{I^{(\text{INT})}} \end{aligned}. \quad (26)$$

The first two terms in the intensities represent the constant background and the last terms show the modulation due to the Φ-dependent interference. Equation (26) quantitatively reveals the main parameters of the real and imaginary gratings formed from the modulation of these intensities. The sum and the difference of the two beams allow the real and imaginary sub-grating profiles to be estimated independently as:

$$\begin{aligned} I_1^{(\text{INT})} - I_2^{(\text{INT})} &= 2E_1E_2\rho\,\text{Im}\left\lfloor\left(\eta_{-1}{}^* + \eta_{+1}\right)e^{i\Phi}\right\rfloor \\ I_1^{(\text{INT})} + I_2^{(\text{INT})} &= 2E_1E_2\rho\,\text{Im}\left[\left(\eta_{-1}{}^* - \eta_{+1}\right)e^{i\Phi}\right] \end{aligned}. \quad (27)$$

By using the relation in equation (12), and normalizing by the DC intensities, we obtain:

$$\frac{I_1^{(\text{INT})} - I_2^{(\text{INT})}}{2\sqrt{I_1^{(\text{DC})}I_2^{(\text{DC})}}} = \rho\Delta\varepsilon_0(1-\xi)\sin\Phi = \rho\, g_{\text{R}}\sin\Phi\,, \quad (28)$$

$$\frac{I_1^{(\text{INT})} + I_2^{(\text{INT})}}{2\sqrt{I_1^{(\text{DC})}I_2^{(\text{DC})}}} = -\rho\Delta\varepsilon_0\xi\cos(\Phi-\delta) = -\rho\, g_{\text{I}}\cos(\Phi-\delta)\,. \quad (29)$$

Here, new symbols g_{R} and g_{I} denote modulation amplitudes of the real and imaginary dielectric function profiles, respectively. Therefore, the difference and sum of the intensities directly yields the real and imaginary modulation amplitudes of the complex dielectric function with δ indicating the phase difference between the real and imaginary gratings.

In Supplementary Fig. 4, we show the measured sum- and difference-intensity profiles corresponding to equations (28) and (29) as interferograms containing information on g_{R}, g_{I} and δ. The polarization parameters of the pump fields are $a_1 = 0$, $a_2 = 0.23$, $\psi_1 = -55°$ and $\psi_2 = 45°$. The sum and difference interferograms produced by PZT-mirror scanning are obtained at the balance factor $\xi = 0.5$. As equations (28) and (29) are applicable for the TE-polarized probe, we monitor only TE components

by including a linear polarizer in front of the photodetectors. The measured interferograms during PZT-mirror scanning are provided in Supplementary Fig. 4(a). As described by the model, the intensities are changing due to the constructed complex grating. However, g_R, g_I and δ are slowly deviating from their original values as the PZT-mirror scanning deforms the original grating profiles. The modulation amplitude factors g_R and g_I determined by equations (28) and (29) for every period of the measured interferograms in Supplementary Fig. 4(a) are plotted in Supplementary Fig. 4(b) as blue and red dots. Similarly the estimated δ is plotted in Supplementary Fig. 4(c). In our experimental set-up, we measure the complex dielectric function at the pump field wavelength. Hence the measured g_I should be compensated by the absorbance ratio of probe and pump wavelength whereas the measured g_R can be taken directly for the probe wavelength. Finally, to estimate the profiles of real and imaginary dielectric function at the beginning of the PZT-mirror scanning, we fit the measured $\Delta\varepsilon_R$, $\Delta\varepsilon_I$ and δ in Supplementary Figs. 4(b) and 4(c) to single exponential functions and reasonably infer g_R, g_I and δ values at $t = 0$ from the fitted curves.

Supplementary Note 4: Reconfiguration of complex lattices with multiple write-erase-rewrite cycles

Establishing the reconfigurability of complex photonic lattices due to our proposed method, we perform a time-domain measurement of diffraction efficiencies $I_{\pm1}$ and contrast ratio under multiple write-erase-rewrite cycles on a single sample spot. The results are shown in Supplementary Fig. 5. In this experiment, pump intensity is fixed at 1.7 W cm^{-2} and the pump-polarization parameters are given in the caption. Supplementary Fig. 5(a) shows temporal profiles of pump intensities for the two pump beams. Strong transient noise in the pump intensity for the initial time range < 200 s is due to fluctuation of the Nd:YAG laser during stabilization. We set the pump-1 intensity to be switched on/off with a regular shutter with sub-ms switching speed while the pump-2 intensity remains constant. Slight modulation of the pump-2 intensity coincident with the pump-1 switching is due to a weak diffraction of pump 1 toward the photodetector monitoring pump 2. This configuration is intended to make the pump-2 beam erase surface-relief grating (real sub-lattice) component during pump 1 is turned off. In addition, different ψ_1 values are applied for each pump-1 on/off cycle. Therefore, the series of the pump-1 on/off cycles produces multiple write-erase-rewrite (WER) cycles for different complex lattice configurations on a single azo-polymer sample spot. Measured $I_{\pm1}$ and corresponding contrast ratio profiles are shown in Supplementary Figs. 5(b) and 5(c). Although period of WER cycles are uneven in a range of 50 s ~ 160 s since we controlled ψ_1 rotation and shutter on/off status manually, it is clearly confirmed that the diffraction intensities are stabilized within 40 s corresponding to the time for the surface-relief grating formation with pump intensity of 1.7 W cm^{-2}. Importantly, the contras ratio profile in Supplementary Fig. 5(c) shows a bell-shaped envelope in response to the change in ψ_1. This confirms that the generated complex lattice configuration on a single sample spot changes with the pump polarization parameters.

Supplementary References

1. Yager, K. G. & Barrett, C. J. All-optical patterning of azo polymer films. *Curr. Opin. Solid State Mater. Sci.* **5,** 487–494 (2001).
2. Natansohn, A. & Rochon, P. Photoinduced Motions in Azo-Containing Polymers. *Chem. Rev.* **102,** 4139–4176 (2002).
3. Xie, S., Natansohn, A. & Rochon, P. Recent developments in aromatic azo polymers research. *Chem. Mater.* (1993).
4. Stumpe, J., Geue, T., Fischer, T. & Menzel, H. Photo-orientation in LB multilayers of amphotropic polymers. *Thin Solid Films* (1996).
5. Wu, Y., Demachi, Y. & Tsutsumi, O. Photoinduced alignment of polymer liquid crystals containing azobenzene moieties in the side chain. 1. Effect of light intensity on alignment behavior. *Macromolecules* **31,** 349–354 (1998).
6. Natansohn, A., Rochon, P., Gosselin, J. & Xie, S. Azo polymers for reversible optical storage. 1. Poly ethylamino]-4-nitroazobenzene]. *Macromolecules* **25,** 2268–2273 (1992).
7. Kumar, J. *et al.* Gradient force: The mechanism for surface relief grating formation in azobenzene functionalized polymers. *Appl. Phys. Lett.* **72,** 2096 (1998).
8. Bian, S., Williams, J. & Kim, D. Photoinduced surface deformations on azobenzene polymer films. *J. Appl. Phys.* **86,** 4498–4508 (1999).
9. Sutter, K. & Günter, P. Photorefractive gratings in the organic crystal 2-cyclooctylamino-5-nitropyridine doped with 7,7,8,8-tetracyanoquinodimethane. *J. Opt. Soc. Am. B* **7,** 2274 (1990).
10. Walsh, C. a. & Moerner, W. E. Two-beam coupling measurements of grating phase in a photorefractive polymer. *J. Opt. Soc. Am. B* **9,** 1642 (1992).
